# Comparing Left Ventricular Diastolic Function between Peritoneal Dialysis and Non-Dialysis Patients with Stage 5 Chronic Kidney Disease: A Propensity Score-Matched Analysis

**DOI:** 10.3390/jcm12155092

**Published:** 2023-08-03

**Authors:** Byoung-Geun Han, Jae Hee Seol, Sooyeon Choi, Donghui Shin, Jae-Seok Kim, Yong Hyuk Kim

**Affiliations:** 1 Department of Nephrology, Yonsei University Wonju College of Medicine, Wonju 26426, Republic of Korea; neptune@yonsei.ac.kr (B.-G.H.);; 2 Department of Pediatrics, Yonsei University Wonju College of Medicine, Wonju 26426, Republic of Korea

**Keywords:** chronic kidney disease, continuous ambulatory peritoneal dialysis, fluid balance, impedance, left ventricular diastolic dysfunction

## Abstract

Patients with chronic kidney disease (CKD) have a high incidence of left ventricular diastolic dysfunction (LVDD), which increases the risk of heart failure and mortality. We assessed fluid overload as an independent risk factor for LVDD in patients with decreased kidney function and compared its impact on the E/e′ ratio as a parameter for assessing left ventricular diastolic functions between patients undergoing continuous ambulatory peritoneal dialysis (CAPD) and those with non-dialysis CKD stage 5 (CKD5) using propensity score matching (PSM). After PSM, 222 patients (CAPD, *n* = 111; CKD5, *n* = 111) were included. Fluid balance was assessed using bio-impedance spectroscopy and LVDD was determined by echocardiography based on an E/e′ ratio of >15. The CKD5 group had a significantly higher E/e′ ratio (*p* = 0.002), while fluid overload (OH/ECW) did not differ significantly between the groups. In the CAPD group, there were no significant differences in OH/ECW between patients with and without LVDD (*p* = 0.517). However, in the CKD5 group, patients with LVDD showed a significantly higher OH/ECW (*p* = 0.001). In a regression analysis investigating factors associated with the E/e′ ratio, OH/ECW was not significantly associated with the E/e′ ratio in the CAPD group (*p* = 0.087), but in the CKD5 group, it was independently correlated (*p* = 0.047). The factors closely associated with LVDD varied depending on dialysis dependence. While fluid overload independently influenced LVDD in non-dialysis patients, it was not statistically significant in patients with CAPD. Early assessment and management of volume status are crucial in addressing LVDD in patients with advanced-stage CKD.

## 1. Introduction

Left ventricular diastolic dysfunction (LVDD) is highly prevalent in the general population and has been associated with an increased risk of cardiovascular events and mortality [1,2,3]. Patients with chronic kidney disease (CKD) have a higher risk of developing cardiovascular disease (CVD) than the general population. Moreover, cardiovascular complications are the leading cause of morbidity and mortality in patients with end-stage renal disease (ESRD) [4]. LVDD is also commonly observed in CKD, irrespective of the cause, and is associated with an elevated risk of heart failure and mortality [5,6,7].

Previous studies have established that age, hypertension, obesity, coronary artery disease, and diabetes are risk factors for LVDD [8,9,10,11]. However, the pathophysiology of CKD-associated cardiomyopathy is highly complex and involves both traditional and non-traditional CKD-related risk factors [12]. Even patients with mild to moderate renal impairment exhibit structural and functional changes consistent with CKD-associated cardiomyopathy. While blood pressure plays a significant role in left ventricular (LV) remodeling in CKD, other CKD-related factors, such as chronic inflammation, elevated levels of calcium and phosphorus, uremic toxins, anemia, fluid overload, vascular calcification, and high serum fibroblast growth factor 23 levels, have also been implicated in the structural and functional changes of the heart in patients with CKD [13]. Although LVDD is strongly associated with increased LV mass and LV hypertrophy (LVH) [14], our previous studies suggested that fluid overload could be an independent risk factor for LVDD in patients with non-dialysis stage 5 CKD (CKD5) [15,16]. Understanding the complex pathophysiology of CKD-associated cardiomyopathy and the role of fluid overload may contribute to the development of targeted interventions for the prevention and management of LVDD in patients with impaired kidney function.

The proper assessment and management of fluid balance play critical roles in promoting hemodynamic stability, reducing cardiovascular complications including LVH, and mitigating the risk of cardiovascular events in patients with CKD [17]. Fluid overload represents a significant step in the pathophysiological pathways leading to acute and/or chronic heart failure in patients with ESRD [18]. Therefore, the accurate and timely assessment of fluid balance is crucial not only for short-term volume control, but also for the long-term prevention of CVD.

Therefore, the primary objective of this study was to investigate the role of fluid overload as an independent risk factor for LVDD in patients with decreased kidney function without intrinsic heart disease. Additionally, we aimed to compare the impact of fluid overload on the E/e′ ratio, an index of diastolic function, between patients undergoing peritoneal dialysis and those with CKD5, using propensity score matching (PSM).

## 2. Materials and Methods

### 2.1. Patients and Data Collection

**Figure 1:**
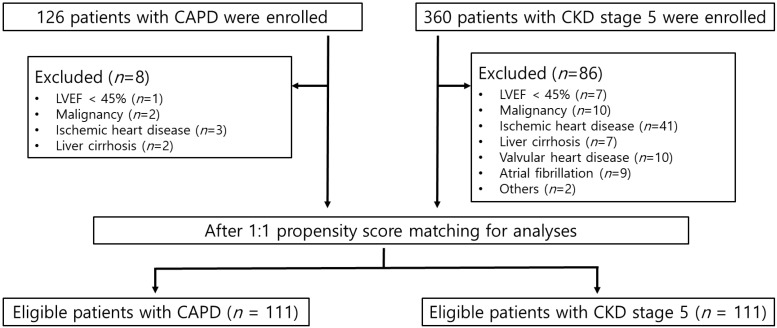
Flow diagram of patient selection in this study. CAPD, continuous ambulatory peritoneal dialysis; CKD, chronic kidney disease; LVEF, left ventricular ejection fraction.

Since 2014, we have registered consecutive patients with CKD5 in a bio-impedance cohort after receiving approval (no. CR316024) from the Institutional Review Board of Yonsei University Wonju Severance Christian Hospital. All the patients provided written informed consent before participating in the study. The CKD5 group consisted only of dialysis-naïve patients. While the CKD5 group was prospectively recruited, all patients in the continuous ambulatory peritoneal dialysis (CAPD) group were included based on a review of their medical records. All patients who underwent bio-impedance spectroscopy (BIS), echocardiography, and laboratory evaluations simultaneously were included in the analysis. Therefore, this was a retrospective observational study. Initially, 126 and 360 patients were assigned to the CAPD and CKD5 groups, respectively. We excluded patients with structural and functional cardiac abnormalities to reduce the effects of underlying heart diseases that could cause LVDD. Patients with a history of angina or myocardial infarction and those with findings of infarction on electrocardiography or regional wall motion abnormalities on echocardiography were excluded. Patients diagnosed with malignancy, liver cirrhosis, ejection fraction less than 45%, cardiac rhythm abnormalities, or valvular heart disease were also excluded. After applying the exclusion criteria, 392 patients (CAPD, *n* = 118; CKD5, *n* = 274) were included. PSM was conducted to minimize the effects of confounding variables. After PSM, 222 patients (111 in each group) were included in the final analysis (Figure 1). The study was conducted in accordance with the principles of the Declaration of Helsinki.

### 2.2. Conventional Echocardiographic Study

Comprehensive echocardiographic measurements were performed using a 3-MHz transducer and commercial ultrasound system (GE Vivid E9; GE Healthcare, Chicago, IL, USA). The imaging procedures followed standard techniques, including M-mode, two-dimensional, and Doppler measurements, in accordance with the guidelines provided by the American Society of Echocardiography and European Association of Cardiovascular Imaging [19,20]. Transmitral inflow velocities were measured using pulsed-wave Doppler in the apical four-chamber view with the sample volume placed at the mitral valve leaflet tips. The transmitral early diastolic (E-wave) velocities were measured. Tissue Doppler imaging in the apical four-chamber view was used to measure the LV myocardial velocities, with the sample volume placed at the septal mitral annulus. We measured the peak early (e′) diastolic mitral annular velocity and calculated the E/e′ ratio. Because E/e′ ratio >15 is typically indicative of elevated LV filling pressure, we used this cutoff value to determine with or without LVDD. The left atrial (LA) dimensions were measured by 2D-guided M-mode echocardiography using the parasternal short-axis view at the base of the heart. The LA volume can be computed using the area-length approximation: LA volume = [8/(3π)] [(A1 × A2)/L], where A1 and A2 are the corresponding LA areas measured in the apical two- and four-chamber views, respectively. LA length L is defined as the shortest of the two long axes measured in each view. The LA volume index (LAVI) was calculated by dividing the LA volume by the body surface area (BSA) using the formula: BSA = 0.007184 × weight^0.425^ × height^0.725^ (m^2^). LV mass was calculated using the following equation: LV mass = 0.8 × {1.04 × ([IVS + LVID + PWT]^3^ − [LVID]^3^)} + 0.6 g, where IVS is the interventricular septum, LVID is the LV internal diameter, and PWT is the inferolateral wall thickness. All measurements were performed at the end-diastole. To correct for BSA, the left ventricular mass index (LVMI) was calculated by dividing the LV mass by BSA. The LV end-diastolic volume (LVEDV) and LV ejection fraction (LVEF) were measured using the biplane-modified Simpson’s rule, according to previously mentioned recommendations. Trained cardiologists who were blinded to the patients’ clinical information performed the echocardiography procedures.

### 2.3. Assessment of the Volume Status

Whole-body BIS was performed using Body Composition Monitoring^TM^ (BCM, Fresenius Medical Care AG & Co., Bad Homburg Vor der Höhe, Germany) to evaluate the body fluid balance. The BCM utilizes alternating electric currents across 50 different frequencies over a range of 5–1000 kHz and measures the impedance of each current. Disposable electrode patches placed on the wrist and ankle were used for all measurements. The validity of the BIS in the general and dialysis populations has been demonstrated in comparison to the gold standard methods. Extracellular water (ECW), intracellular water, and total body water (TBW) were automatically provided by the onboard software of the BCM device using equations based on the Hanai mixture theory developed by Moissl et al. [21]. The three-compartment BIS model separates body weight into normally hydrated lean tissue mass, normally hydrated adipose tissue mass, and extracellular fluid overload, commonly represented as the volume of overhydration (OH) [22]. The OH, presented as a positive or negative value, can be calculated from the difference between the actual measured and expected ECW [23]. In the case of peritoneal dialysis, the BCM device was applied after the peritoneal cavity was emptied. As the clinical significance of OH values varies based on patient body size, relative overhydration (OH/ECW) was primarily used to determine the volume status for the analysis. Furthermore, the amount of fluid in the body increases in obese individuals, and this increase is more prominent in the extracellular compartment [24]. Therefore, the ratio of ECW to TBW (ECW/TBW) might have been overestimated in determining volume status of patients. To address this issue, this study used OH/ECW instead of ECW/TBW.

### 2.4. Statistical Analysis

Patient characteristics were summarized using means and standard deviations for continuous variables, and frequencies and percentages for categorical variables. For PSM, we selected variables that affected the LV diastolic function and fluid balance as covariates. The potential confounders included age, sex, prevalence of diabetes, systolic blood pressure, diastolic blood pressure, and serum albumin levels. Using nearest-neighbor matching, we matched the patients in a 1:1 ratio without replacement. The standardized mean differences between the groups were calculated to confirm whether matching was effective by setting the caliper at a width of 0.2. The R (version 3.6.0, The R Foundation for Statistical Computing, Vienna, Austria) package ‘table one 0.13.2’ ‘Matching 4.10-8’ was used.

After PSM, the CAPD group and the CKD5 group were categorized into two distinct groups, determined by their E/e′ ratio: E/e′ ratio > 15 and E/e′ ratio ≤ 15, denoting the presence or absence of LVDD. Differences in the clinical variables between the two groups of patients were tested using two-sample *t*-tests for continuous variables. Nominal variables were compared using chi-square and Fisher’s exact tests, as appropriate. Pearson correlation coefficients were calculated to evaluate the associations of E/e′ ratio with other variables, such as laboratory findings, echocardiographic parameters, and a marker of volume status. We performed a multiple linear regression analysis using variables based on previous reports, such as age, sex, diabetes, LVMI, and OH/ECW, to assess independent variables associated with the E/e′ ratio. Statistical analyses were performed using IBM SPSS Statistics (version 25.0; IBM, Armonk, NY, USA). Statistical significance was set at *p*-values < 0.05.

## 3. Results

After excluding patients who did not meet the inclusion criteria, the total number of patients was 392 (118 with CAPD and 274 with CKD5) (Supplementary Table S1). Patients with fluid overload (OH/ECW ≥ 15%) were observed in 26 CAPD patients and 123 CKD5 patients, showing a statistically significant difference (*p* < 0.001). Additionally, 15 CAPD patients and 103 CKD5 patients had LVDD with an E/e′ ratio > 15; the differences were statistically significant (*p* < 0.001). After PSM (*n* = 222), the levels of high-sensitivity C-reactive protein, intact parathyroid hormone, hemoglobin, total protein, total cholesterol, serum calcium, serum phosphorus, and body mass index (BMI) were significantly different between the two groups. OH/ECW did not show any significant differences between the two groups. However, the E/e′ ratio was significantly higher in the CKD5 group (*p* = 0.002) (Supplementary Table S2).

**Figure 2:**
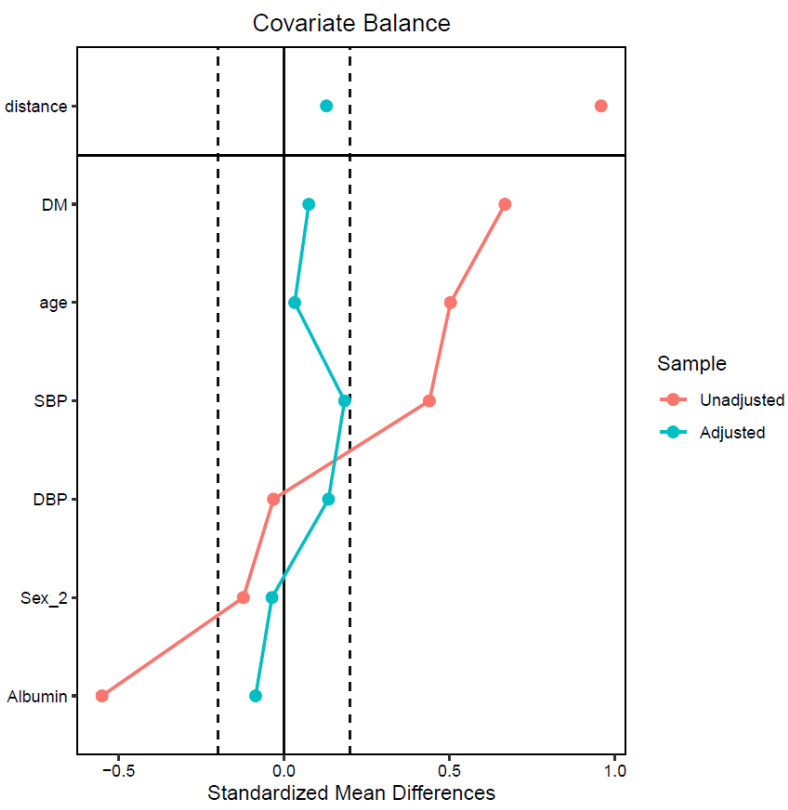
Covariate balance measured by standardized mean differences. DBP, diastolic blood pressure; DM, diabetes mellitus; SBP, systolic blood pressure.

Table 1 presents the results before and after PSM. After PSM, the caliper refers to the decrease in the maximum allowable difference to <20% (0.2) of the standardized mean difference between the two groups for all baseline covariates (Figure 2) [25].

After PSM, a significant association was found between the E/e′ ratio and various factors, including systolic blood pressure, left atrial diameter (LAD), LAVI, LVEDV, LVMI, BMI, hemoglobin, total protein, albumin, serum calcium, and OH/ECW (Table 2).

The clinical characteristics of each group categorized according to the presence or absence of LVDD are presented in Table 3. After PSM, 15 patients (13.5%) in the CAPD group and 27 (24.3%) in the CKD5 group exhibited LVDD. In the CAPD group, there were no significant differences in the OH/ECW between patients with and without LVDD (*p* = 0.591). In addition, echocardiographic parameters, including LAD, LAVI, LVEDV, LVMI, and LVEF, were not significantly different. In contrast, in the CKD5 group, patients with LVDD showed a markedly higher OH/ECW (*p* = 0.002). Among the echocardiographic parameters, the LAVI and LVMI were significantly higher in patients with LVDD. Interestingly, patients with LVDD in both groups were more likely to have a history of diabetes.

We initially conducted linear regression analyses using variables based on previous reports, such as age, sex, prevalent diabetes, LVMI, and OH/ECW, in the entire patient population after performing PSM to identify potential factors associated with the E/e′ ratio. Sex, diabetes, and LVMI were significantly associated with the E/e′ ratio. OH/ECW did not show a significant association in the overall patient population; however, there were differences in the association of OH/ECW depending on the presence or absence of renal replacement therapy. To identify the factors that differed between the two groups, linear regression analyses were performed by dividing each group into additional analyses. In the CAPD group, there were no significant changes in meaningful parameters; in the CKD5 group, sex was not significant, and OH/ECW emerged as a new independent factor with statistical significance (*p* = 0.047) (Table 4).

## 4. Discussion

CVD is the most common cause of death in patients with advanced CKD [26]. LVDD in patients with pre-dialysis CKD is significantly linked to worse cardiovascular outcomes and higher all-cause mortality rates [27,28]. Known risk factors for LVDD include age, hypertension, diabetes, ischemic coronary disease, obesity, and LVH. Meanwhile, uremic toxins, chronic inflammation, vascular calcification, anemia, and fluid overload are also known to be associated with LVDD in patients with ESRD [13,29]. Although the factors associated with LVDD differed depending on whether the participants had dialysis-dependent CKD, LVDD can occur even in the early stages of CKD and potentially lead to heart failure with preserved ejection fraction [30,31]. The development of LVDD is associated with a declining estimated glomerular filtration rate (eGFR). As eGFR decreases, the prevalence of LVDD in patients with the lowest eGFR (<45 mL/min/1.73 m^2^) is close to 51% by year 5 [31]. In our previous study, the prevalence of LVDD in CKD5 was 45.2% [16]. Before PSM, the CAPD group had a prevalence of 12.7%, whereas that in the CKD5 group was 37.6%; the difference between the two groups was statistically significant (Supplementary Table S1).

Previous studies on the impact of different dialysis methods (hemodialysis and peritoneal dialysis) on echocardiographic parameters have yielded heterogeneous and contradictory results. Moreover, few studies have compared the structural and functional changes in the myocardium between patients undergoing dialysis and those with advanced-stage CKD who were not on dialysis. Therefore, to investigate the differences between patients with CAPD and CKD5, we performed a PSM analysis. In the actual analysis, it was necessary to minimize the influence of confounders. One of these was LVMI. In CKD, both an increase in afterload, represented by hypertension and arteriosclerosis, and an increase in preload, induced by anemia and fluid overload, are associated with LVH. Traditionally, LVH has been implicated in the development of diastolic dysfunction in patients with CKD [32]. Furthermore, LVMI was an independent determinant of LVDD in patients undergoing hemodialysis. The prevalence of LVH was higher in patients undergoing conventional hemodialysis than in those undergoing peritoneal dialysis patients [33]. Therefore, we included peritoneal dialysis patients, and in our regression analysis for each group, LVMI was found to be a significant independent factor.

Another confounding factor is the presence or absence of diabetes. Diabetes is a metabolic condition that contributes to LVDD development [34,35,36]. In our study, diabetes was identified as a significant independent factor associated with LVDD in both groups, which is consistent with a previous report highlighting its contribution to LVDD.

It has been suggested that sex-specific risk factors, such as hormonal changes, increase the risk of LVDD and subsequent heart failure [37]. However, previous studies based on community-based cohort data have shown no significant sex differences in the prevalence of LVDD [38]. In our study, there were no significant differences in the prevalence of LVDD between males and females in either group (Table 3). However, in the CAPD group, there was an independent association between LVDD and sex; in the CKD5 group, there was a marginal *p*-value indicating a potential association (Table 4). We were unable to perform additional analyses because we did not measure hormones, such as estrogen.

When peritoneal dialysis is initiated, a substantial number of patients are already experiencing a state of fluid overload. [39]. However, in a study investigating the utility of BIS-guided fluid management in patients undergoing peritoneal dialysis, there were no significant differences in echocardiographic parameters, such as LVMI and E/e′ ratio, when fluid control was effectively managed regardless of the use of BIS [40]. Fluid overload has indirect effects on the cardiovascular system and is associated with malnutrition and/or inflammation, leading to an increased risk of cardiovascular events [41]. In our analysis, we observed a higher degree of fluid overload in patients with LVDD in the CKD5 group, whereas no significant difference in volume status was noted in the CAPD group (Table 3). Furthermore, in the CAPD group, OH/ECW ratio was not a significant independent factor associated with LVDD (Table 4). These findings indirectly suggest that effective volume control positively affects LVDD. If fluid control is effectively managed in dialysis patients, other risk factors such as hyperphosphatemia may play a more prominent role in the development of LVDD [42]. In contrast to our study, it has been reported that arterial function and diabetes were associated with the E/e′ ratio in non-dialysis patients, and volume overload and anemia were associated with the E/e′ ratio in dialysis patients, respectively [30]. Notably, the patient characteristics and methods utilized to assess fluid balance in our study differed from those described in the aforementioned report. Therefore, the main determinants of the E/e′ ratio may vary between dialysis and non-dialysis patients owing to differences in patient characteristics.

This study has several limitations. First, the formal definition of LVDD includes echocardiographic measurements beyond the E/e′ ratio [20]. However, in line with other studies, a simplified and modified definition of LVDD (i.e., E/e′ ratio > 15) was employed in this study. Second, causality cannot be assessed because our study did not investigate the sequential changes in E/e′ ratio associated with the removal of fluid overload through renal replacement therapy in this observational study. Considering that our patients presented with a complex combination of diabetes, hypertension, and kidney disease, along with the use of multiple medications, an important clinical aspect would be to assess longitudinal changes in cardiac dysfunction in relation to disease progression and treatment modalities. Notably, fluid overload could be an aggravating factor of LVDD in patients with stages 4–5. Therefore, implementing tailored treatment strategies targeting this risk factor prior to dialysis initiation may prevent LV remodeling and improve patient outcomes. Third, when utilizing a single measurement, there is a possibility of bias, and the results may not fully capture the fluctuations in fluid status that could occur over time. Consequently, this approach may lead to an underestimation or overestimation of the true relationship between fluid overload and LVDD. Lastly, although the duration of dialysis treatment may influence myocardial remodeling in patients with CAPD, it was not considered in the analysis as one of the confounders. Under the assumption of relatively similar conditions, the concept of dialysis treatment duration could not be applied to the CKD5 group for comparison with the CAPD group.

However, this study has several strengths. Previously, Moré et al. reported that patients with a history of heart failure exhibited a significantly higher prevalence of cardiovascular risk factors and comorbidities, along with a worse prognosis, even in individuals with non-dialysis CKD stages 4–5 [43]. In contrast, all patients in our study were free of intrinsic heart disease and represented a relatively homogeneous population in terms of eGFR. Therefore, it is worth noting that there were differences in the independent factors related to LVDD between the groups receiving and those not receiving renal replacement therapy.

In conclusion, our findings indicate that the factors closely associated with LVDD may vary depending on whether the participants have dialysis-dependent CKD. Specifically, fluid overload has emerged as a significant independent factor in patients with CKD who are not on dialysis. These observations highlight the importance of addressing fluid overload as a potential therapeutic target in pre-dialysis patients with CKD to mitigate the risk of LVDD development.

## Figures and Tables

**Table 1:** Baseline characteristics before and after propensity score matching.

	Before Propensity Score Matching			After Propensity Score Matching		
	CAPD	CKD5	SMD	CAPD	CKD5	SMD
No. of patients	118	274		111	111	
Age, years	52.40 ± 13.06	59.28 ± 13.69	0.50	53.75 ± 12.12	54.19 ± 13.60	0.03
Sex	1.49	1.43	−0.12	1.50	1.48	−0.04
Diabetes	0.31	0.64	0.67	0.33	0.37	0.07
SBP, mmHg	134.36	142.92	0.44	133.56	137.12	0.18
DBP, mmHg	81.02	80.65	−0.03	80.42	81.96	0.13
Albumin, g/dL	3.80	3.50	−0.55	3.79	3.74	−0.09

CAPD, continuous ambulatory peritoneal dialysis; CKD5, chronic kidney disease stage 5; DBP, diastolic blood pressure; SBP, systolic blood pressure; SMD, standardized mean difference.

**Table 2:** Correlation analysis of E/e′ ratio with clinical and bio-impedance spectroscopy parameters after matching.

Variables	Total (*n* = 222)		CAPD (*n* = 111)		CKD5 (*n* = 111)	
	Correlation Coefficient	*p*-Value	Correlation Coefficient	*p*-Value	Correlation Coefficient	*p*-Value
Age, years	0.093	0.168	0.127	0.183	0.062	0.520
SBP, mmHg	0.235	<0.001	0.141	0.139	0.302	0.001
DBP, mmHg	0.053	0.433	−0.018	0.850	0.093	0.329
BMI, kg/m^2^	0.152	0.023	0.118	0.219	0.097	0.309
LAD, cm	0.182	0.007	0.093	0.330	0.306	0.001
LAVI, mL/m^2^	0.345	<0.001	0.362	<0.001	0.394	<0.001
LVEDV, mL	0.252	<0.001	0.159	0.105	0.231	0.015
LVMI, g/m^2^	0.325	<0.001	0.219	0.021	0.409	<0.001
LVEF, %	−0.097	0.151	−0.235	0.014	0.036	0.709
hs-CRP, mg/dL	0.085	0.213	0.219	0.021	0.020	0.836
iPTH, pg/mL	−0.032	0.639	−0.151	0.118	−0.003	0.973
Hemoglobin, g/dL	−0.148	0.028	−0.037	0.697	−0.069	0.470
Total protein, g/dL	−0.169	0.012	−0.046	0.628	−0.184	0.053
Albumin, g/dL	−0.176	0.009	−0.129	0.177	−0.197	0.039
Calcium, mg/dL	−0.162	0.016	0.051	0.597	−0.178	0.061
Phosphorus, mg/dL	0.139	0.093	0.060	0.534	0.114	0.233
OH/ECW, %	0.291	<0.001	0.147	0.124	0.399	<0.001

BMI, body mass index; CAPD, continuous ambulatory peritoneal dialysis; CKD5, chronic kidney disease stage 5; DBP, diastolic blood pressure; ECW, extracellular water; hs-CRP, high-sensitivity C-reactive protein; iPTH, intact parathyroid hormone; LAD, left atrial dimension; LAVI, left atrial volume index; LVEF, left ventricular ejection fraction; LVEDV, left ventricular end-diastolic volume; LVMI, left ventricular mass index; OH, overhydration; SBP, systolic blood pressure.

**Table 3:** Comparison of demographics, serum chemistry, echocardiographic findings, and volume status between patients with and without left ventricular diastolic dysfunction in each group.

Variables		CAPD (*n* = 111)			CKD5 (*n* = 111)		
		E/e′ Ratio ≤ 15 (*n* = 96)	E/e′ Ratio > 15 (*n* = 15)	*p*-Value	E/e′ Ratio ≤ 15 (*n* = 84)	E/e′ Ratio > 15 (*n* = 27)	*p*-Value
E/e′ ratio		9.8672 ± 2.65	19.38 ± 4.52	<0.001	10.82 ± 2.76	19.37 ± 3.84	<0.001
Age, years		53.01 ± 12.48	58.47 ± 8.43	0.105	54.35 ± 13.87	53.70 ± 12.99	0.832
Sex	Male	50 (89.3%)	6 (10.7%)	0.384	45 (77.6%)	13 (22.4%)	0.624
	Female	46 (83.6%)	9 (16.4%)		39 (73.6%)	14 (26.4%)	
Diabetes	Yes	27 (73.0%)	10 (27.0%)	0.003	24 (58.5%)	17 (41.5%)	0.001
	No	69 (93.2%)	5 (6.8%)		60 (85.7%)	10 (14.3%)	
OH/ECW	<15%	74 (85.1%)	13 (14.9%)	0.517	70 (83.3%)	14 (16.7%)	0.001
	≥15%	22 (91.7%)	2 (8.3%)		14 (51.9%)	13 (48.1%)	
SBP, mmHg		132.61 ± 22.66	139.60 ± 17.50	0.257	135.24 ± 20.78	143.00 ± 22.40	0.100
DBP, mmHg		80.76 ± 12.64	78.27 ± 10.41	0.469	82.04 ± 13.29	81.74 ± 11.85	0.918
BMI, kg/m^2^		22.97 ± 3.16	24.52 ± 4.06	0.094	25.03 ± 4.19	25.33 ± 3.45	0.735
LAD, cm		4.48 ± 0.51	4.54 ± 0.49	0.613	4.38 ± 0.46	4.58 ± 0.44	0.053
LAVI, mL/m^2^		35.51 ± 14.52	43.39 ± 17.80	0.078	33.91 ± 7.94	40.85 ± 13.65	0.017
LVEDV, mL		98.86 ± 44.02	107.26 ± 52.10	0.518	139.94 ± 25.59	150.07 ± 28.36	0.084
LVMI, g/m^2^		101.90 ± 28.23	112.60 ± 28.49	0.176	104.07 ± 24.72	120.41 ± 32.33	0.021
LVEF, %		62.43 ± 5.14	60.58 ± 10.67	0.520	62.66 ± 5.12	61.63 ± 5.48	0.375
hs-CRP, mg/dL		0.19 ± 0.40	0.55 ± 1.00	0.192	0.90 ± 2.35	0.54 ± 1.14	0.475
iPTH, pg/mL		289.28 ± 214.61	181.56 ± 119.29	0.010	347.09 ± 254.10	342.21 ± 164.83	0.926
Hemoglobin, g/dL		10.60 ± 1.46	10.49 ± 1.34	0.777	9.08 ± 1.27	8.97 ± 0.80	0.602
Total protein, g/dL		6.64 ± 0.55	6.56 ± 0.68	0.631	6.36 ± 0.72	6.15 ± 0.80	0.211
Albumin, g/dL		3.80 ± 0.35	3.70 ± 0.36	0.305	3.76 ± 0.49	3.66 ± 0.52	0.363
Calcium, mg/dL		8.84 ± 0.89	8.71 ± 0.87	0.615	7.89 ± 1.21	7.75 ± 1.14	0.612
Phosphorus, mg/dL		5.16 ± 1.14	5.00 ± 1.09	0.603	5.79 ± 1.55	5.92 ± 1.13	0.692
OH/ECW, %		9.22 ± 10.07	10.14 ± 5.24	0.591	6.38 ± 13.03	17.46 ± 15.66	0.002

BMI, body mass index; CAPD, continuous ambulatory peritoneal dialysis; CKD5, chronic kidney disease stage 5; DBP, diastolic blood pressure; ECW, extracellular water; hs-CRP, high-sensitivity C-reactive protein; iPTH, intact parathyroid hormone; LAD, left atrial dimension; LAVI, left atrial volume index; LVEF, left ventricular ejection fraction; LVEDV, left ventricular end-diastolic volume; LVMI, left ventricular mass index; OH, overhydration; SBP, systolic blood pressure.

**Table 4:** Factors independently associated with E/e′ ratio as a dependent variable.

	Total (*n* = 222)		CAPD (*n* = 111)		CKD5 (*n* = 111)	
	B (95% CI)	*p*-Value	B (95% CI)	*p*-Value	B (95% CI)	*p*-Value
Group	1.616 (0.551, 2.682)	0.003				
OH/ECW, %	0.044 (−0.006, 0.095)	0.087	−0.015 (−0.109, 0.078)	0.744	0.064 (0.001, 0.126)	0.047
Age, years	0.031 (−0.011, 0.073)	0.151	0.035 (−0.109, 0.078)	0.289	0.025 (−0.032, 0.083)	0.387
Sex	2.145 (1.042, 3.247)	<0.001	2.729 (1.087, 4.370)	0.001	1.481 (−0.055, 3.017)	0.059
Diabetes	2.583 (1.312, 3.853)	<0.001	3.180 (1.303, 5.058)	0.001	2.372 (0.594, 4.151)	0.009
LVMI, g/m^2^	0.053 (0.032, 0.073)	<0.001	0.049 (0.020, 0.078)	0.001	0.060 (0.031, 0.090)	<0.001

B, β coefficient; CAPD, continuous ambulatory peritoneal dialysis; CI, confidence interval; CKD5, chronic kidney disease stage 5; ECW, extracellular water; LVMI, left ventricular mass index; OH, overhydration.

## Data Availability

Data cannot be made publicly available due to ethical concerns, as it is not possible to anonymise data sufficient for public access.

## Supplementary Materials

The following supporting information can be downloaded at: https://www.mdpi.com/article/10.3390/jcm12155092/s1, Table S1: Comparison of variables between groups before propensity score matching; Table S2: Comparison of demographics, serum chemistry, echocardiographic findings, and volume status between groups after propensity score matching.

## References

[1] Kuznetsova T., Herbots L., Lopez B., Jin Y., Richart T., Thijs L., Gonzalez A., Herregods M.C., Fagard R.H., Diez J. (2009). Prevalence of left ventricular diastolic dysfunction in a general population. Circ. Heart Fail..

[2] Mogelvang R., Sogaard P., Pedersen S.A., Olsen N.T., Marott J.L., Schnohr P., Goetze J.P., Jensen J.S. (2009). Cardiac dysfunction assessed by echocardiographic tissue Doppler imaging is an independent predictor of mortality in the general population. Circulation.

[3] Kuznetsova T., Thijs L., Knez J., Herbots L., Zhang Z., Staessen J.A. (2014). Prognostic value of left ventricular diastolic dysfunction in a general population. J. Am. Heart Assoc..

[4] Lai A.C., Bienstock S.W., Sharma R., Skorecki K., Beerkens F., Samtani R., Coyle A., Kim T., Baber U., Camaj A. (2021). A personalized approach to chronic kidney disease and cardiovascular disease: JACC review topic of the week. J. Am. Coll. Cardiol..

[5] Farshid A., Pathak R., Shadbolt B., Arnolda L., Talaulikar G. (2013). Diastolic function is a strong predictor of mortality in patients with chronic kidney disease. BMC Nephrol..

[6] Otsuka T., Suzuki M., Yoshikawa H., Sugi K. (2009). Left ventricular diastolic dysfunction in the early stage of chronic kidney disease. J. Cardiol..

[7] Park M., Hsu C.Y., Li Y.M., Mishra R.K., Keane M., Rosas S.E., Dries D., Xie D.W., Chen J., He J. (2012). Associations between kidney function and subclinical cardiac abnormalities in CKD. J. Am. Soc. Nephrol..

[8] Carvalho J.C., Farand P., Do H.D., Brochu M.C., Bonenfant F., Lepage S. (2013). Effect of age and sex on echocardiographic left ventricular diastolic function parameters in patients with preserved ejection fraction and normal valvular function. Cardiol. J..

[9] Russo C., Jin Z., Homma S., Rundek T., Elkind M.S., Sacco R.L., Di Tullio M.R. (2010). Effect of diabetes and hypertension on left ventricular diastolic function in a high-risk population without evidence of heart disease. Eur. J. Heart Fail..

[10] Russo C., Jin Z., Homma S., Rundek T., Elkind M.S., Sacco R.L., Di Tullio M.R. (2011). Effect of obesity and overweight on left ventricular diastolic function: A community-based study in an elderly cohort. J. Am. Coll. Cardiol..

[11] Maragiannis D., Schutt R.C., Gramze N.L., Chaikriangkrai K., McGregor K., Chin K., Nabi F., Little S.H., Nagueh S.F., Chang S.M. (2015). Association of left ventricular diastolic dysfunction with subclinical coronary atherosclerotic disease burden using coronary artery calcium scoring. J. Atheroscler. Thromb..

[12] Law J.P., Pickup L., Pavlovic D., Townend J.N., Ferro C.J. (2023). Hypertension and cardiomyopathy associated with chronic kidney disease: Epidemiology, pathogenesis and treatment considerations. J. Hum. Hypertens..

[13] Escoli R., Carvalho M.J., Cabrita A., Rodrigues A. (2019). Diastolic dysfunction, an underestimated new callenge in dalysis. Ther. Apher. Dial..

[14] Ogawa T., Nitta K. (2018). Clinical impact of left ventricular diastolic dysfunction in chronic kidney disease. Contrib. Nephrol..

[15] Han B.G., Lee J.Y., Kim M.R., Shin H., Kim J.S., Yang J.W., Kim J.Y. (2020). Fluid overload is a determinant for cardiac structural and functional impairments in type 2 diabetes mellitus and chronic kidney disease stage 5 not undergoing dialysis. PLoS ONE.

[16] Kim J.S., Yang J.W., Yoo J.S., Choi S.O., Han B.G. (2017). Association between E/e´ ratio and fluid overload in patients with predialysis chronic kidney disease. PLoS ONE.

[17] Tsai Y.C., Chiu Y.W., Tsai J.C., Kuo H.T., Hung C.C., Hwang S.J., Chen T.H., Kuo M.C., Chen H.C. (2015). Association of fluid overload with cardiovascular morbidity and all-cause mortality in stages 4 and 5 CKD. Clin. J. Am. Soc. Nephrol..

[18] Tuegel C., Bansal N. (2017). Heart failure in patients with kidney disease. Heart.

[19] Lang R.M., Badano L.P., Mor-Avi V., Afilalo J., Armstrong A., Ernande L., Flachskampf F.A., Foster E., Goldstein S.A., Kuznetsova T. (2015). Recommendations for cardiac chamber quantification by echocardiography in adults: An update from the American Society of Echocardiography and the European Association of Cardiovascular Imaging. J. Am. Soc. Echocardiogr..

[20] Nagueh S.F., Smiseth O.A., Appleton C.P., Byrd B.F., Dokainish H., Edvardsen T., Flachskampf F.A., Gillebert T.C., Klein A.L., Lancellotti P. (2016). Recommendations for the evaluation of left ventricular diastolic function by echocardiography: An update from the American Society of Echocardiography and the European Association of Cardiovascular Imaging. J. Am. Soc. Echocardiogr..

[21] Moissl U.M., Wabel P., Chamney P.W., Bosaeus I., Levin N.W., Bosy-Westphal A., Korth O., Muller M.J., Ellegard L., Malmros V. (2006). Body fluid volume determination via body composition spectroscopy in health and disease. Physiol. Meas..

[22] Chamney P.W., Wabel P., Moissl U.M., Muller M.J., Bosy-Westphal A., Korth O., Fuller N.J. (2007). A whole-body model to distinguish excess fluid from the hydration of major body tissues. Am. J. Clin. Nutr..

[23] Wabel P., Chamney P., Moissl U., Jirka T. (2009). Importance of whole-body bioimpedance spectroscopy for the management of fluid balance. Blood Purif..

[24] Waki M., Kral J.G., Mazariegos M., Wang J., Pierson R.N., Heymsfield S.B. (1991). Relative expansion of extracellular fluid in obese vs. nonobese women. Am. J. Physiol..

[25] Austin P.C. (2011). Optimal caliper widths for propensity-score matching when estimating differences in means and differences in proportions in observational studies. Pharm. Stat..

[26] Thompson S., James M., Wiebe N., Hemmelgarn B., Manns B., Klarenbach S., Tonelli M. (2015). Cause of death in patients with reduced kidney function. J. Am. Soc. Nephrol..

[27] Suh S.H., Oh T.R., Choi H.S., Kim C.S., Bae E.H., Oh K.H., Choi K.H., Oh Y.K., Ma S.K., Kim S.W. (2022). Association of left ventricular diastolic dysfunction with cardiovascular outcomes in patients with pre-dialysis chronic kidney disease: Findings from KNOW-CKD study. Front. Cardiovasc. Med..

[28] Kim M.K., Kim B., Lee J.Y., Kim J.S., Han B.G., Choi S.O., Yang J.W. (2013). Tissue Doppler-derived E/e′ ratio as a parameter for assessing diastolic heart failure and as a predictor of mortality in patients with chronic kidney disease. Korean J. Intern. Med..

[29] Ogawa T., Koeda M., Nitta K. (2015). Left ventricular diastolic dysfunction in end-stage kidney disease: Pathogenesis, diagnosis, and treatment. Ther. Apher. Dial..

[30] Hsu H.C., Norton G.R., Robinson C., Woodiwiss A.J., Dessein P.H. (2021). Potential determinants of the E/e’ ratio in non-dialysis compared with dialysis patients. Nephrology.

[31] Miyajima Y., Toyama T., Mori M., Nakade Y., Sato K., Yamamura Y., Ogura H., Yoneda-Nakagawa S., Oshima M., Miyagawa T. (2021). Relationships between kidney dysfunction and left ventricular diastolic dysfunction: A hospital-based retrospective study. J. Nephrol..

[32] Wang X., Shapiro J.I. (2019). Evolving concepts in the pathogenesis of uraemic cardiomyopathy. Nat. Rev. Nephrol..

[33] Kimura H., Takeda K., Turuya K., Mukai H., Muto Y., Okuda H., Furusho M., Ueno T., Nakashita S., Miura S. (2011). Left ventricular mass index is an independent determinant of diastolic dysfunction in patients on chronic hemodialysis: A tissue Doppler imaging study. Nephron Clin. Pract..

[34] Choi S.R., Lee Y.K., Park H.C., Kim D.H., Cho A., Kang M.K., Choi S. (2021). Clinical significance of central systolic blood pressure in LV diastolic dysfunction and CV mortality. PLoS ONE.

[35] Okura H. (2020). Subclinical diastolic dysfunction in diabetes: How to detect, how to manage?. Eur. Heart J. Cardiovasc. Imaging.

[36] Bouthoorn S., Valstar G.B., Gohar A., den Ruijter H.M., Reitsma H.B., Hoes A.W., Rutten F.H. (2018). The prevalence of left ventricular diastolic dysfunction and heart failure with preserved ejection fraction in men and women with type 2 diabetes: A systematic review and meta-analysis. Diab. Vasc. Dis. Res..

[37] Zhao Z., Wang H., Jessup J.A., Lindsey S.H., Chappell M.C., Groban L. (2014). Role of estrogen in diastolic dysfunction. Am. J. Physiol. Heart Circ. Physiol..

[38] van Ommen A., Canto E.D., Cramer M.J., Rutten F.H., Onland-Moret N.C., Ruijter H.M.D. (2022). Diastolic dysfunction and sex-specific progression to HFpEF: Current gaps in knowledge and future directions. BMC Med..

[39] Ronco C., Verger C., Crepaldi C., Pham J., De Los Rios T., Gauly A., Wabel P., Van Biesen W., Ipod-Pd Study Group (2015). Baseline hydration status in incident peritoneal dialysis patients: The initiative of patient outcomes in dialysis (IPOD-PD study). Nephrol. Dial. Transplant..

[40] Oh K.H., Baek S.H., Joo K.W., Kim D.K., Kim Y.S., Kim S., Oh Y.K., Han B.G., Chang J.H., Chung W. (2018). Does routine bioimpedance-guided fluid management provide additional benefit to non-anuric peritoneal dialysis patients? Results from COMPASS clinical trial. Perit. Dial. Int..

[41] Dekker M.J.E., van der Sande F.M., van den Berghe F., Leunissen K.M.L., Kooman J.P. (2018). Fluid overload and inflammation axis. Blood Purif..

[42] Ye M., Tian N., Liu Y.Q., Li W., Lin H., Fan R., Li C.L., Liu D.H., Yao F.J. (2016). High serum phosphorus level is associated with left ventricular diastolic dysfunction in peritoneal dialysis patients. PLoS ONE.

[43] Valdivielso More S., Vicente Elcano M., Garcia Alonso A., Pascual Sanchez S., Galceran Herrera I., Barbosa Puig F., Belarte-Tornero L.C., Ruiz-Bustillo S., Morales Murillo R.O., Barrios C. (2023). Characteristics of patients with heart failure and advanced chronic kidney disease (Stages 4–5) not undergoing renal replacement therapy (ERCA-IC Study). J. Clin. Med..

