# Supplementary material for: Comparing Left Ventricular Diastolic Function between Peritoneal Dialysis and Non-Dialysis Patients with Stage 5 Chronic Kidney Disease: A Propensity Score-Matched Analysis
Source: J Clin Med. 2023 Aug 3;12(15):5092. doi: 10.3390/jcm12155092 (PMC10420270; doi:10.3390/jcm12155092)
Supplement: Supplementary file 1 [file jcm-12-05092-s001.zip › jcm-2514526-supplementary.pdf]

Supplementary Table S1. Comparison of variables between groups before propensity score matching.

Variables		Total (N = 392)	CAPD (n = 118)	CKD5 (n = 274)	*P*-value
Age, years		57.21 ± 13.85	52.40 ± 13.06	59.28 ± 13.69	<0.001
Sex	Male	216 (55.1%)	60 (27.8%)	156 (72.2%)	0.266
	Female	176 (44.9%)	58 (33.0%)	118 (67.0%)	
Diabetes	Yes	211 (53.8%)	37 (17.5%)	174 (82.5%)	<0.001
	No	181 (46.2%)	81 (44.8%)	100 (55.2%)	
OH/ECW	<15%	243 (62.0%)	92 (78.0%)	151 (55.1%)	<0.001
	≥15%	149 (38.0%)	26 (22.0%)	123 (44.9%)	
LVDD	⩽15	274 (69.9%)	103 (87.3%)	171 (62.4%)	<0.001
	>15	118 (30.1%)	15 (12.7%)	103 (37.6%)	
SBP, mmHg		140.34 ± 20.65	134.36 ± 22.05	142.92 ± 19.50	<0.001
DBP, mmHg		80.76 ± 11.79	81.02 ± 12.56	80.65 ± 11.47	0.780
BMI, kg/m^2		24.45 ± 3.91	23.17 ± 3.28	24.99 ± 4.04	<0.001
LAD, cm		4.53 ± 0.49	4.50 ± 0.50	4.54 ± 0.49	0.429
LAVI, mL/m^2		37.61 ± 12.11	37.09 ± 15.19	37.82 ± 10.66	0.647
E/e′ ratio		13.48 ± 5.30	10.94 ± 4.32	14.58 ± 5.31	<0.001
LVEDV, mL		130.49 ± 38.49	101.71 ± 44.95	142.25 ± 28.09	<0.001
LVMI, g/m^2		110.32 ± 27.14	104.07 ± 28.75	113.00 ± 26.00	0.003
LVEF, %		62.61 ± 5.60	62.10 ± 6.06	62.82 ± 5.39	0.252
hs-CRP, mg/dL		0.83 ± 2.30	0.24 ± 0.52	1.10 ± 2.71	<0.001
iPTH, pg/mL		300.58 ± 217.97	282.30 ± 211.54	308.30 ± 220.56	0.284
Hemoglobin, g/dL		9.48 ± 1.49	10.54 ± 1.44	9.02 ± 1.26	<0.001
Total protein, g/dL		6.25 ± 0.77	6.62 ± 0.58	6.09 ± 0.79	<0.001
Albumin, g/dL		3.59 ± 0.52	3.80 ± 0.36	3.50 ± 0.56	<0.001
Total cholesterol, mg/dL		153.76 ± 45.81	169.45 ± 42.83	147.00 ± 45.47	<0.001
HDL-C, mg/dL		42.07 ± 15.12	50.28 ± 16.69	38.47 ± 12.85	<0.001
LDL-C, mg/dL		86.23 ± 37.64	95.37 ± 33.19	82.26 ± 38.80	0.002
Triglyceride, mg/dL		131.75 ± 67.80	131.98 ± 63.02	131.65 ± 69.84	0.965
Alkaline phosphatase, U/L		80.87 ± 38.93	79.23 ± 35.13	81.57 ± 40.50	0.585

Calcium, mg/dL	8.04 ± 1.16	8.80 ± 0.89	7.72 ± 1.10	<0.001
Phosphorus, mg/dL	5.73 ± 1.51	5.23 ± 1.17	5.95 ± 1.58	<0.001
OH/ECW, %	13.37 ± 13.69	9.37 ± 9.35	15.09 ± 14.87	<0.001

BMI, body mass index; CAPD, continuous ambulatory peritoneal dialysis; CKD5, chronic kidney disease stage 5; DBP, diastolic blood pressure; ECW, extracellular water; HDL-C, high-density lipoprotein cholesterol; hs-CRP, high-sensitivity C-reactive protein; iPTH, intact parathyroid hormone; LAD, left atrial dimension; LAVI, left atrial volume index; LDL-C, low-density lipoprotein cholesterol; LVEF, left ventricular ejection fraction; LVEDV, left ventricular end-diastolic volume; LVDD, left ventricular diastolic dysfunction; LVMI, left ventricular mass index; OH, overhydration; SBP, systolic blood pressure.

Supplementary Table S2. Comparison of demographics, serum chemistry, echocardiographic findings, and volume status between groups after propensity score matching.

Variables		Total (N = 222)	CAPD (n = 111)	CKD5 (n = 111)	*P*-value
Age, years		53.97 ± 12.86	53.75 ± 12.12	54.19 ± 13.60	0.799
Sex	Male	114 (51.4%)	56 (49.1%)	58 (50.9%)	0.788
	Female	108 (48.6%)	55 (50.9%)	53 (49.1%)	
Diabetes	Yes	78 (35.1%)	74 (51.4%)	70 (48.6%)	0.574
	No	144 (64.9%)	37 (47.4%)	41 (52.6%)	
OH/ECW	<15%	171 (77.0%)	87 (50.9%)	84 (49.1%)	0.632
	≥15%	51 (23.0%)	24 (47.1%)	27 (52.9%)	
LVDD	≤15	180 (81.1%)	96 (53.3%)	84 (46.7%)	0.040
	>15	42 (18.9%)	15 (35.7%)	27 (64.3%)	
SBP, mmHg		135.34 ± 21.75	133.56 ± 22.10	137.13 ± 21.35	0.222
DBP, mmHg		81.19 ± 12.62	80.42 ± 12.35	81.96 ± 12.90	0.364
BMI, kg/m^2		24.14 ± 3.79	23.18 ± 3.32	25.11 ± 4.01	<0.001
LAD, cm		4.46 ± 0.48	4.49 ± 0.51	4.43 ± 0.46	0.398
LAVI, mL/m^2		36.03 ± 12.70	36.50 ± 15.10	35.60 ± 10.03	0.603
E/e´ ratio		11.94 ± 4.68	10.98 ± 4.40	12.90 ± 4.78	0.002
LVEDV, mL		121.78 ± 42.32	99.98 ± 45.00	142.41 ± 26.52	<0.001
LVMI, g/m^2		105.70 ± 27.99	103.35 ± 28.38	108.05 ± 27.53	0.212
LVEF, %		62.29 ± 5.69	62.17 ± 6.17	62.41 ± 5.20	0.761
hs-CRP, mg/dL		0.53 ± 1.57	0.24 ± 0.53	0.82 ± 2.15	0.008
iPTH, pg/mL		311.09 ± 224.10	275.32 ± 207.56	345.90 ± 234.83	0.019
Hemoglobin, g/dL		9.82 ± 1.52	10.59 ± 1.44	9.05 ± 1.17	<0.001

Total protein, g/dL	6.47 ± 0.68	6.62 ± 0.57	6.31 ± 0.75	<0.001
Albumin, g/dL	3.76 ± 0.43	3.79 ± 0.35	3.74 ± 0.50	0.407
Total cholesterol, mg/dL	158.40 ± 48.35	169.27 ± 43.84	147.53 ± 50.36	0.001
HDL-C, mg/dL	44.27 ± 15.68	49.35 ± 16.51	39.10 ± 12.95	<0.001
LDL-C, mg/dL	87.89 ± 39.30	95.69 ± 33.83	80.02 ± 42.88	0.003
Triglyceride, mg/dL	136.57 ± 77.62	135.26 ± 63.16	137.86 ± 89.85	0.804
Alkaline phosphatase, U/L	78.58 ± 32.45	79.79 ± 35.88	77.37 ± 28.71	0.579
Calcium, mg/dL	8.34 ± 1.15	8.82 ± 0.88	7.85 ± 1.19	<0.001
Phosphorus, mg/dL	5.48 ± 1.34	5.14 ± 1.13	5.83 ± 1.46	<0.001
OH/ECW, %	9.21 ± 12.22	9.34 ± 9.55	9.08 ± 14.45	0.872

BMI, body mass index; CAPD, continuous ambulatory peritoneal dialysis; CKD5, chronic kidney disease stage 5; DBP, diastolic blood pressure; ECW, extracellular water; HDL-C, high-density lipoprotein cholesterol; hs-CRP, high-sensitivity C-reactive protein; iPTH, intact parathyroid hormone; LAD, left atrial dimension; LAVI, left atrial volume index; LDL-C, low-density lipoprotein cholesterol; LVEF, left ventricular ejection fraction; LVEDV, left ventricular end-diastolic volume; LVDD, left ventricular diastolic dysfunction; LVMI, left ventricular mass index; OH, overhydration; SBP, systolic blood pressure.
